# Differences in Endothelial Activation and Dysfunction Induced by Antiphospholipid Antibodies Among Groups of Patients With Thrombotic, Refractory, and Non-refractory Antiphospholipid Syndrome

**DOI:** 10.3389/fphys.2021.764702

**Published:** 2021-12-02

**Authors:** Manuela Velásquez, Luisa F. Peláez, Mauricio Rojas, Raúl Narváez-Sánchez, Jesús A. Velásquez, Carlos Escudero, Sebastián San Martín, Ángela P. Cadavid

**Affiliations:** ^1^Grupo Reproducción, Departamento de Microbiología y Parasitología, Facultad de Medicina, Universidad de Antioquia UdeA, Medellín, Colombia; ^2^Grupo de Inmunología Celular e Inmunogenética, Facultad de Medicina, Universidad de Antioquia UdeA, Unidad de Citometría de Flujo, Sede de Investigación Universitaria, Medellín, Colombia; ^3^Physiology and Biochemistry Research Group-PHYSIS, Faculty of Medicine, University of Antioquia UdeA, Medellín, Colombia; ^4^Hospital Universitario San Vicente Fundación, Medellín, Colombia; ^5^Red Iberoamericana de Alteraciones Vasculares Asociadas a TRanstornos del EMbarazo (RIVATREM), Chillán, Chile; ^6^Vascular Physiology Laboratory, Basic Sciences Department, Faculty of Sciences, Universidad del Bio-Bio, Chillán, Chile; ^7^Group of Research and Innovation in Vascular Health (GRIVAS Health), Chillán, Chile; ^8^Biomedical Research Center School of Medicine, Universidad de Valparaíso, Valparaíso, Chile; ^9^Grupo de Investigación en Trombosis, Facultad de Medicina, Universidad de Antioquia UdeA, Medellín, Colombia

**Keywords:** antiphospholipid syndrome, endothelial cells, endothelial activation and dysfunction, beta 2-glycoprotein I, immunoglobulin G, antiphosholipid syndrome

## Abstract

Antiphospholipid syndrome (APS) is an autoimmune disorder characterized by pregnancy morbidity or thrombosis and persistent antiphospholipid antibodies (aPL) that bind to the endothelium and induce endothelial activation, which is evidenced by the expression of adhesion molecules and the production of reactive oxygen species (ROS) and subsequent endothelial dysfunction marked by a decrease in the synthesis and release of nitric oxide (NO). These endothelial alterations are the key components for the development of severe pathological processes in APS. Patients with APS can be grouped according to the presence of other autoimmune diseases (secondary APS), thrombosis alone (thrombotic APS), pregnancy morbidity (obstetric APS), and refractoriness to conventional treatment regimens (refractory APS). Typically, patients with severe and refractory obstetric APS exhibit thrombosis and are classified as those having primary or secondary APS. The elucidation of the mechanisms underlying these alterations according to the different groups of patients with APS could help establish new therapies, particularly necessary for severe and refractory cases. Therefore, this study aimed to evaluate the differences in endothelial activation and dysfunction induced by aPL between patients with refractory obstetric APS and other APS clinical manifestations. Human umbilical vein endothelial cells (HUVECs) were stimulated with polyclonal immunoglobulin-G (IgG) from different groups of patients *n* = 21), including those with primary (VTI) and secondary thrombotic APS (VTII) and refractory primary (RI+), refractory secondary (RII+), and non-refractory primary (NR+) obstetric APS. All of them with thrombosis. The expression of adhesion molecules; the production of ROS, NO, vascular endothelial growth factor (VEGF), and endothelin-1; and the generation of microparticles were used to evaluate endothelial activation and dysfunction. VTI IgG induced the expression of adhesion molecules and the generation of microparticles and VEGF. RI+ IgG induced the expression of adhesion molecules and decreased NO production. RII+ IgG increased the production of microparticles, ROS, and endothelin-1 and reduced NO release. NR+ IgG increased the production of microparticles and endothelin-1 and decreased the production of VEGF and NO. These findings reveal differences in endothelial activation and dysfunction among groups of patients with APS, which should be considered in future studies to evaluate new therapies, especially in refractory cases.

## Introduction

The endothelium is a cell monolayer that lines the lumen of the lymphatic and blood vessels with paracrine, endocrine, and autocrine functions to control vascular remodeling and tone, blood flow, and leukocyte trafficking (Kruger-Genge et al., 2019). In diseases such as antiphospholipid syndrome (APS), endothelial activation is induced, thus producing proinflammatory and procoagulant molecules, leading to alterations in vascular tone, coagulability, and endothelial dysfunction (Liao, 2013; Corban et al., 2017; Miranda et al., 2019). APS is an autoimmune disease characterized by clinical manifestations of thrombosis or pregnancy morbidity and persistent antiphospholipid antibodies (aPL), including lupus anticoagulant (LA), anti-cardiolipin antibodies (aCL), and anti-β2-glycoprotein-I (aβ2GPI; Miyakis et al., 2006). The association between endothelial activation and dysfunction and the clinical manifestations of patients with APS is unclear (Velásquez et al., 2018).

Patients with APS present different clinical manifestations, aPL profile immunoglobulin (Ig) isotype, titers, and medication response. Patients with obstetric APS who only have pregnancy morbidity and are repeatedly positive for at least one of the aPL have a successful pregnancy in 75% of the cases if they receive heparin and aspirin (ASA) as standard treatment (Schreiber and Hunt, 2019). Contrarily, patients with obstetric APS who have pregnancy morbidity plus thrombosis and high aPL titers, particularly those with triple aPL positivity, have an ineffective standard of care, but the addition of hydroxychloroquine (HCQ) improves the gestational outcome (De Carolis et al., 2017; Ruffatti et al., 2017; Mekinian et al., 2018). aPL in these patients lead to endothelial activation and dysfunction, which deteriorate vascular relaxation through diverse mechanisms, including (1) the production of reactive oxygen species (ROS); (2) decreased bioavailability of nitric oxide (NO); (3) enhanced synthesis of vasoconstrictor factors, such as endothelin-1; (4) adhesion molecule synthesis; and (5) the release of endothelial microparticles (Mayer-Pickel et al., 2016; Engel et al., 2017; Sacharidou et al., 2018; Miranda et al., 2019; Velasquez et al., 2019; Alvarez et al., 2021). These pathological effects are induced by beta 2-glycoprotein-I (β2GPI) bound to the endothelium, but this mechanism is unclear.

Endothelial dysfunction is marked by a decrease in the synthesis and release of NO from the endothelium (Cyr et al., 2020). NO downregulates the interaction with leukocytes via a decreased expression of adhesion molecules (Gao et al., 2017). However, adhesion molecules and ROS are upregulated under endothelial dysfunction, generating a vicious circle of deterioration in NO availability (Forstermann et al., 2017; Uthman et al., 2019). Therefore, aPL are endothelial NO production antagonists, and NO reduction contributes to thrombi formation and leukocyte adhesion (Ramesh et al., 2011). However, the differences in aPL effect on different groups of patients, especially the refractory cases, are unclear.

Among the factors listed earlier, endothelial microparticles are 0.1–1 μm vesicles, presenting a procoagulant activity (Holnthoner et al., 2017). In cardiovascular disorders, an increase in the production of endothelial microparticles is detected by labeling with CD31 and annexin V, suggesting that these particles have the role as an endothelial dysfunction biomarker (Deng et al., 2017; Leite et al., 2020). However, its difference in producing a different profile of microparticles in different groups of patients or its potential consequences on endothelial dysfunction is unclear.

Thus, the literature described aPL-mediated endothelial activation and dysfunction via an increase in the expression of adhesion molecules, oxidative stress, microparticle generation, and a decrease in NO, and little is known about the participation of β2GPI in these processes. Additionally, the different clinical characteristics of patients with APS in modifying aPL-mediated endothelial dysfunction are unclear. Therefore, this study aimed to evaluate the differences in endothelial activation and dysfunction induced by aPL between patients with refractory obstetric APS and other APS clinical manifestations. Moreover, the participation of β2GPI in aPL-mediated endothelial dysfunction was further explored. This knowledge would provide tools to evaluate new therapeutic strategies in refractory or more severe APS cases.

## Materials and Methods

### Study Subjects

A total of 41 female patients were included in this study: 21 diagnosed with APS and 20 in the control group. According to clinical characteristics, female patients with obstetric APS and thrombosis (*n* = 11) were divided as follows: refractory primary (RI+, *n* = 3), refractory secondary [with systemic lupus erythematosus (SLE)] (RII+, *n* = 2), and non-refractory primary (NR+, *n* = 6). Refractoriness was defined as obstetric manifestations as patients manifest a new episode of pregnancy morbidity despite optimal pharmacological treatment with heparin and ASA during pregnancy (Mekinian et al., 2017). Patients with vascular thrombosis (*n* = 10) were classified as primary (VTI, *n* = 6) and secondary with SLE (VTII, *n* = 4). The control group included patients who were negative for aPL and with clinical manifestations of thrombosis (*n* = 10), classified as primary (patients who were negative for aPL with thrombosis without autoimmune disease (VTI/aPL*-*, *n* = 5) or secondary with SLE (patients who were negative for aPL with thrombosis and SLE (VTII/aPL*-*, *n* = 5) and patients with previous uncomplicated pregnancies [normal human serum (NHS), *n* = 10]. Exclusion criteria for all study participants were other associated diseases such as infections, diabetes, cancer, or chronic disease other than systemic autoimmune diseases due to the inclusion of patients with secondary APS. Patients were recruited from the Recurrent Pregnancy Loss Program of Reproduction Group (University of Antioquia) and Anticoagulation Clinic (San Vicente Fundación Hospital), with the previous approval of the Ethics Committee from the Medical Research Institute-School of Medicine (University of Antioquia). Informed consent was obtained from each participant. None of the patients were pregnant or presented an acute thrombosis episode when the samples were obtained.

### Reagents, Materials, and Antibodies

Reagents, materials, and antibodies were acquired from the following manufacturers: Limulus amebocyte lysate, NuncTM filter flask, BODIPY C11, MitoSOX, eFluor, and anti-CD31-FITC from Thermo Scientific (Waltham, MA, United States); Type I collagenase from Invitrogen (San Diego, CA, United States); basal endothelial cell culture medium and fetal bovine serum (FBS) from Promocell (Heidelberg, Germany); RPMI-1640, FBS, Opti-MEM, and PBS from Gibco (Grand Island, NY, United States); penicillin and gentamicin from Genfar (Bogotá, Colombia); human-β2GPI from Louisville APL Diagnostics (Louisville, KY, United States); Amphotericin B, PKH67, dichlorofluorescein diacetate (DCFH-DA), propidium iodide, lipopolysaccharide (LPS), ASA, HCQ, vascular endothelial growth factor (VEGF) 165, and NG-nitro-L-arginine methyl ester (L-NAME) from Sigma (St. Louis, MO, United States); anti-VCAM-1-PE and anti-E-selectin-Alexa Fluor 700, from R&D Systems by Bio-techne (Minneapolis, MN, United States); 4-amino-5-methylamino-2′, 7-difluoroflurescein diacetate (DAF-FM-DA) from Cayman Chemical (Ann Arbor, MI, United States); enoxaparin (ENX) from Procaps (Barranquilla, Colombia); and annexin V and 7-aminoactinomycin D (7-AAD) from BD Pharmigen (San Diego, CA, United States).

### Antiphospholipid Antibodies

The Clinical and Laboratory Standards Institute recommendations were followed for plasma LA determination (Pengo et al., 2009). APTT-SP (Instrumentation Laboratory, Orangeburg, NY, United States) was used to demonstrate the antibody dependence on phospholipids. Dilute Russell’s viper venom time (dRVVT) screen and dRVVT confirm (Instrumentation Laboratory, Orangeburg, NY, United States) were used to detect LA. The presence of aCL and aβ2GPI was evaluated using commercial kits (BioSystems, Barcelona, Spain and Human, Wiesbaden, Germany, respectively) in serum and purified immunoglobulin-G (IgG). IgG from the serum pool of patients in each group was purified by affinity chromatography using protein G-Sepharose (General Electric Healthcare, NY, United States), as described (Alvarez et al., 2017). Endotoxins were detected in purified IgG using the Limulus amebocyte lysate assay.

### Human Umbilical Vein Endothelial Cell Isolation

Human umbilical cords were obtained from female patients with uncomplicated pregnancies who attended obstetric services at the Hospital San Vicente Fundación, Medellín, Colombia. Voluntary female patients signed informed consent. The Ethical Committee of the same Hospital approved the sample collection. Human umbilical vein endothelial cells (HUVECs) were isolated by mechanical and enzymatic digestion based on the modified protocol of Jaffe (Jaffe et al., 1973; Gil-Villa et al., 2020). Briefly, the umbilical vein was perfused with a pericranial needle with 5 ml of type I collagenase followed by 20 min of incubation at 37°C. The umbilical vein content was centrifuged, and the button of cells (including HUVECs) was cultured in a NuncTM filter flask with 10 ml of basal endothelial cell culture medium and 2% of FBS. Different umbilical cords from healthy patients were used to isolate HUVECs included in each experiment. HUVECs were used until passage three. In all the experiments, HUVECs were maintained in Opti-MEM in serum-free conditions to perform the stimuli with IgG and human-β2GPI.

### THP-1 Cell Culture

THP-1 cells, derived from a 1-year-old infant with acute monocytic leukemia, were obtained from the American Type Culture Collection (CRL-1593, Manassas, VA, United States) and cultured in RPMI plus 10% FBS and antibiotic solution of 100 IU of penicillin, 50 μg of gentamicin, and 250 ng of amphotericin B at 37°C with 5% CO_2_ and 60% relative humidity.

### Evaluation of Endothelial Activation

#### Model of Adhesion

In 24-well plates, 5 × 10^4^ HUVECs were added per well to evaluate the expression of E-selectin and vascular cell adhesion molecule 1 (VCAM-1) induced by 50 μg/ml IgG for 24 h with or without 5 μg/ml human-β2GPI. Subsequently, the cells were detached by incubating with trypsin for 2 min, deactivated with 2% FBS, and washed with PBS. The following antibodies were added (diluted 1:100): anti-VCAM-1-PE and anti-E-selectin-Alexa Fluor 700. Non-specific bindings were blocked with 20% FBS in PBS. The cells were evaluated using a flow cytometer LSR Fortessa (BD), acquiring 10,000 events per sample. The obtained data were analyzed using the FlowJo^®^ v7.6.2 software. Positive cell values are indicated as the percentage and median fluorescence intensity (MFI). The fluorescence-minus-one (FMO) control included all antibodies with the conjugated fluorochromes except the molecules of interest. The FMO control was prepared for each antibody. Subsequently, the effect of IgG on the monocyte adhesion to the endothelium was detected. THP-1 cells were labeled with the fluorescent dye PKH67 according to the manufacturer’s instructions. Briefly, in 24-well plates, 0.2 μl of PKH67 and 20 μl of diluent C (included with the kit) were added to each 1 × 10^4^ cells per well. After that, 80 μl of FBS was added to remove excess dye, and the cells were incubated with 250 μg/ml of IgG for 24 h. Simultaneously, 1 × 10^4^ HUVECs per well were stimulated with IgG under the same conditions like THP-1. After 24 h of IgG stimulation, THP-1 monocytes were added to the HUVEC monolayer and incubated for 2 h. Finally, the cells were washed with PBS at 37°C to remove non-adherent cells. THP-1 cells attached to the PKH67-labeled endothelium were detected using the spectrofluorometer Varioskan TM LUX multimode microplate reader (Thermo Scientific, Waltham, MA, United States). Photographs were acquired using the DS-Fi1 camera (Nikon, Shinagawa, Japan) adapted to the Axio Vert.A1 fluorescence microscope (Zeiss, Berlín, Germany) with a 20X objective. LPS (4 μg/ml) was included as a positive control for E-selectin and VCAM-1 expression and the THP-1 cell adhesion to the endothelium. All stimuli were performed in Opti-MEM plus antibiotics at 37°C under 5% CO_2_ and 60% relative humidity.

#### Oxidative Stress Evaluation

Oxidative stress was evaluated as an indicator of endothelial activation by the intracellular ROS, superoxide anion (O_2_^–^) production, and lipid peroxidation in aPL-stimulated endothelial cells. In 24-well plates, 5 × 10^4^ HUVECs were added per well and stimulated for 24 h with 250 μg/ml IgG. The cells were detached using trypsin, which was inactivated with Opti-MEM containing 10% FBS. The cells were washed two times by centrifugation at 580 × *g* for 5 min with 600 μl of PBS. To detect ROS production, 0.05 μM of DCFH-DA and 0.5 μM of propidium iodide were added to the cells. Then, 0.825 μM of BODIPY C11 in 1,200 μl of PBS was added to detect membrane lipid peroxidation. For the evaluation of mitochondrial O_2_^–^ production evaluation, 0.02 μM MitoSOX probe with 0.01 μM eFluor was added to the cells. Using the LSR Fortessa flow cytometer (BD), 1 × 10^4^ events per sample were acquired. The obtained data were analyzed using the FlowJo^®^ v7.6.2 program. Values were reported as MFI. Cells were stimulated with 0.5 and 1 mM hydrogen peroxide as an endothelial oxidative stress positive control. About 2, 4, and 8 μg/ml of LPS were used as a positive control to induce O_2_^–^. The cells were incubated with 5 μg/ml of β2GPI to detect the effect of aβ2GPI in IgG treatment.

### Detection of Endothelial Dysfunction

#### Generation of Cell-Derived Endothelial Microparticles *in vitro*

The generation of cell-derived endothelial microparticles *in vitro* was detected based on the Pericleous protocol (Pericleous et al., 2013). Briefly, HUVECs were treated with IgG or 8 μg/ml of LPS for 24 h, and the supernatants were collected and centrifuged at 3,000 × *g* for 5 min to remove debris. The supernatant was centrifuged at 15,000 × *g* for 60 min to obtain the cell-derived endothelial microparticles, which were resuspended in a filtered binding buffer and stained with 1:100 of anti-CD31 and annexin V. Readings were made on a flow cytometer, acquiring total events in 120 s. Cell-derived microparticle size was defined using 0. 5-, 1-, and 2-μm polystyrene beads. The levels of basal and LPS-induced cell-derived endothelial microparticles and the signals from polystyrene beads were differentiated from the electronic noise. The data were analyzed using the FlowJo^®^ v7.6.2 program. A number of events were reported as microparticles.

#### Nitric Oxide Release

Nitric oxide bioavailability was evaluated using the probe DAF-FM-DA in the HUVECs stimulated with aPL. DAF-FM-DA is a cell-permeable probe; once NO crosses the plasma membrane, this dye is deacetylated by intracellular esterases and is transformed into DAF-FM. DAF-FM has a baseline fluorescence of 0.005 but increases to 0.81 (160 times) when it reacts with NO with an excitation/emission maximum of 495/515 nm. In 24-well plates, 5 × 10^4^ HUVECs were added per well and stimulated for 1 h with 250 μg/ml of IgG with or without 5 μg/ml of β2GPI. After incubation with IgG, HUVECs were washed with PBS, and 1 μM of DAF-FM-DA was added, followed by 20 min of incubation at 37°C. Fluorescence of DAF-FM-DA was analyzed using the Varioskan TM LUX multimode microplate reader (Thermo Scientific, Waltham, MA, United States). As positive controls to induce NO release, 100 and 200 ng/ml of VEGF165 were included. As a negative control for NO production, 100 μM of the nitric oxide synthase (NOS) antagonist, L-NAME, was used.

#### Endothelin-1 and Vascular Endothelial Growth Factor Production

This study detected endothelin-1 and VEGF production in supernatants of HUVECs treated (24 h) with IgG with or without β2GPI. Both endothelin-1 (R&D) and VEGF (Invitrogen, Waltham, MA, United States) were measured using human ELISA kits according to the respective manufacturer’s instructions.

### Modulation of Endothelial Dysfunction

Aspirin at 10 mM, ENX at 50 IU/ml, and HCQ at 1 μg/ml were simultaneously added with IgG to detect the modulating effect on NO release.

### Assessment of Cell Viability

The effect of IgG, ASA, ENX, HCQ, and β2GPI on cell viability was evaluated using 7-AAD. The stimuli were performed under the same conditions previously described to detect NO. Following 1-h incubation, the dead cells of 7-AAD+ were detected using an LSR Fortessa flow cytometer (BD). The obtained data were analyzed using the FlowJo^®^ v7.6.2 program and reported as% dead cells. As a positive control of dead cells, 4- and 8-mM hydrogen peroxide (Sigma, St. Louis, MO, United States) were used.

### Statistical Analysis

Data distribution was evaluated using the Shapiro–Wilk tests. Data are expressed as mean ± SD. A *t*-test, Mann–Whitney test and two-way ANOVAII were performed, and the comparison between means was determined using the Holm–Sidak post-test. Statistical analyses were performed using Prism6 (GraphPad Software, Inc., San Diego, CA, United States). For all the cases, the value of *p* < 0.05 was considered significant.

## Results

### Patient Characteristics

No differences were observed in the age of patients of different groups. The samples from patients with VTI, RI+, RII+, and NR+ were positive for aPL in the serum and purified IgG. Contrarily, the group with VTII alone was positive for LA and serum aCL. The RII+ group was positive for aPL; however, it had lower values than patients with refractory and NR+ APS (RI+ and NR+). Patients with RI+ had higher LA values compared with other groups. Two patients in this group had catastrophic APS. The IgG samples of controls (NHS, VTI/aPL*-*, and VTII/aPL*-*) were negative for aPL (Table 1). All the tested IgG samples were negative for endotoxins. For all the cases, secondary APS indicated the presence of SLE. All patients with refractory APS had pregnancy morbidity even after ASA and heparin treatment (Table 1). Different characteristics of thrombotic events were observed among patients with thrombosis from which the received treatment was derived (Table 1).

**TABLE 1:** Characteristics of patients included in this study.

Characteristics	NHS (*n* = 10)	VTI/aPL- (*n* = 5)	VTII/aPL- (*n* = 5)	VTI (*n* = 6)	VTII (*n* = 4)	RI+ (*n* = 3)	RII+ (*n* = 2)	NR+ (*n* = 6)
Age (mean of years ± SD)	35.5 ± 5.46	38.8 ± 15.61	42.8 ± 12.09	30 ± 11.21	35.25 ± 11.95	34.33 ± 6.81	46.5 ± 3.54	38.83 ± 7.22
Pregnancy loss (mean and rank) ≤10 weeks of pregnancy	0	0	0	0	0	2.33 (2-5)	1 (1-2)	1.2 (1-5)
>10 weeks of pregnancy	0	0	0	0	0	2.33 (1-5)	3 (1-5)	0.6 (0-2)
Preeclampsia < 34 weeks (number of patients)	0	0	0	0	0	3	1	2
Intrauterine growth restriction (number of patients)	0	1	0	0	0	2	0	1
Arterial thrombosis (number of patients)	0	0	0	1	0	0	0	0
Deep vein thrombosis (number of patients)	0	3	2	3	4	3	2	6
Pulmonary embolism (number of patients)	0	1	5	3	2	1	1	3
Stroke (number of patients)	0	2	0	1	1	0	1	0
Recurrent thrombosis (number of patients)	0	3	3	2	4	3	2	3
Warfarin (number of patients)	0	3	3	6	4	1	2	3
Prednisolone (number of patients)	0	3	2	0	2	0	1	0
Statins (number of patients)	0	0	0	0	0	0	1	0
Chloroquine (number of patients)	0	2	1	1	1	1	2	2
Hydroxychloroquine (number of patients)	0	0	0	0	1	0	0	0
Lupus anticoagulant (mean ± SD)^†^	1.06 ± 0.05	1.13 ± 0.01	1.10 ± 0.02	2.62 ± 0.16 (+)	2.26 ± 0.6 (+)	3.3 ± 0.24 (+)	1.34 ± 0.24 (+)	1.92 ± 0.27 (+)
IgG anti-β2 glycoprotein I (serum) (U/ml) (mean ± SD)^‡^	2.29 ± 0.07	2.52 ± 0.32	2.08 ± 0.33	56.37 ± 8.79 (+)	5.9 ± 0.44	97.88 ± 14.02 (+)	16.56 ± 5.1 (+)	67.79 ± 32.5 (+)
IgG anti-cardiolipin (serum) (GPL/ml) (mean ± SD)^£^	0	1.08 ± 0.15	0	97.09 ± 6.96 (+)	12.8 ± 0.51 (+)	102.2 ± 15.58 (+)	29.71 ± 1.1 (+)	93.73 ± 2.56 (+)
IgG anti-β2 glycoprotein (mean ± SD)^‡^	0.84 ± 0.01	0.59 ± 0.08	0.72 ± 0.38	14.09 ± 21.54 (+)	3.95 ± 3.9	21.78 ± 25.96 (+)	7.9 ± 6.6 (+)	38.55 ± 32.43 (+)
IgG anti-cardiolipin (mean ± SD)^£^	0	0	0	10.34 ± 1.95 (+)	3.93 ± 0.87	34.7 ± 1.82 (+)	16.54 ± 0.5 (+)	80.03 ± 1.63 (+)

*GPL [immunoglobulin-G (IgG) phospholipid units]. IgG anti-β2-glycoprotein and IgG anti-cardiolipin were evaluated in 250 μg/ml of purified IgG.*

*^†^Values greater than 1.2 indicate a positive test for lupus anticoagulant (LA).*

*^‡^Values greater than 7 U/ml anti-β2-glycoprotein-I are positive.*

*^£^Values greater than 10 GPL/ml anti-cardiolipin are positive.*

### Immunoglobulin-G From Patients With Primary Thrombotic Antiphospholipid Syndrome and RI+ Induced an Increase in Expression of Adhesion Molecule as an Endothelial Activation Indicator

**FIGURE 1:**
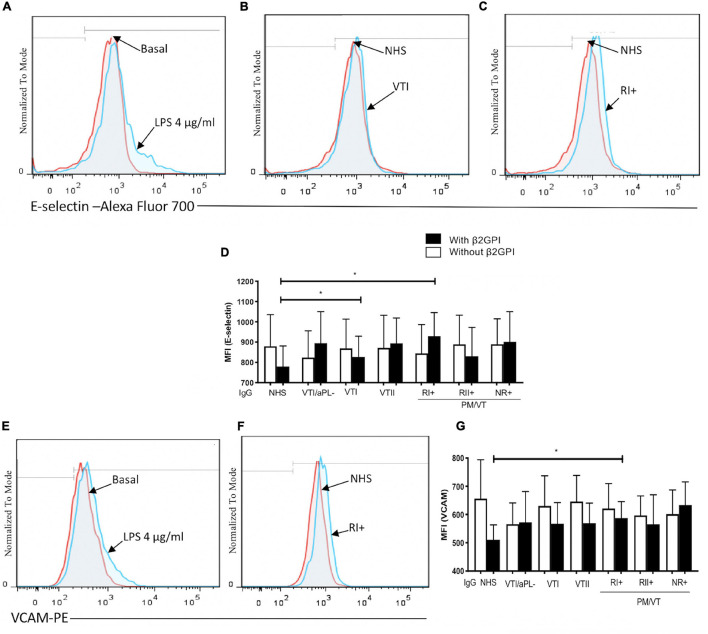
Immunoglobulin-G (IgG) from patients with primary antiphospholipid syndrome (APS) [primary thrombotic APS (VTI) and refractory primary obstetric APS (RI+)] induced an increase in the adhesion molecule expression in human umbilical vein endothelial cells (HUVECs). **(A,E)** Lipopolysaccharide (LPS) at a concentration of 4 μg/ml was included as a positive control, and the increased E-selectin and vascular cell adhesion molecule 1 (VCAM-1) expression was compared with unstimulated cells. **(B–D)** The median fluorescence intensity (MFI) of E-selectin increased in HUVECs stimulated with beta 2-glycoprotein-I (β2GPI) and IgG from patients with VTI and RI+ compared with the IgG/normal human serum (NHS) control. **(F,G)** The MFI of VCAM-1 increased in HUVECs stimulated with β2GPI and IgG from patients with RI+ compared with the IgG/NHS control. A two-way ANOVA and the Holm–Sidak post-test (*p < 0.05 and **p < 0.01) were performed. The results were obtained from three independent experiments. **(A–C,E,G)** Representative histograms. PM/VT, pregnancy morbidity and vascular thrombosis.

In all the experiments, LPS increased E-selectin and VCAM-1 expressions (Figures 1A,E). In β2GPI-stimulated endothelial cells, the VTI and RI+ group IgG increased E-selectin compared with NHS control IgG (Figures 1B–D). Also, in β2GPI-stimulated endothelial cells, RI+ IgG increased VCAM-1 in contrast with the NHS control IgG (Figures 1F,G).

### Immunoglobulin-G From Patients With RI+ Induced an Increased Monocyte Adhesion

**FIGURE 2:**
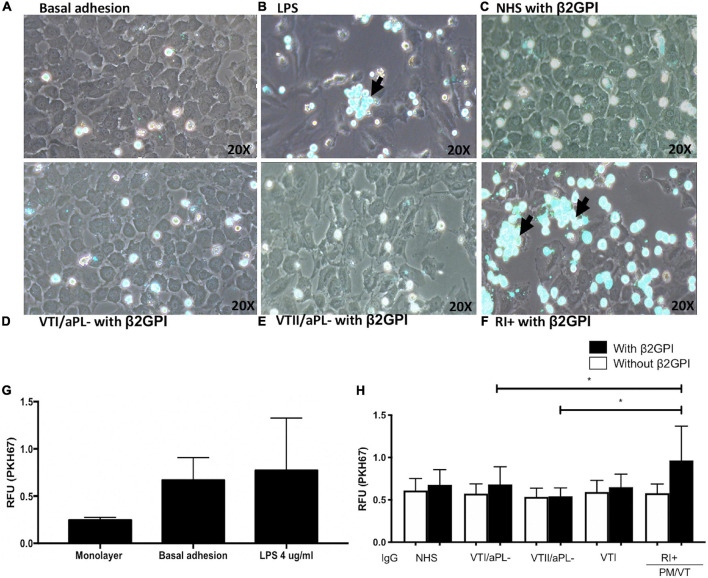
Immunoglobulin-G from patients with refractory and primary obstetric APS (RI+) induces increased monocyte adhesion to the endothelium. **(A)** Baseline adhesion. **(B)** LPS. The adhesion of monocytes to the endothelium in cells stimulated with β2GPI plus: **(C)** IgG/NHS; **(D)** IgG/patients negative for aPL with thrombosis without autoimmune disease (VTI/aPL-); **(E)** IgG/patients negative for aPL with thrombosis and SLE (VTII/aPL-); and **(F)** IgG/RI+. **(A–F)** Representative images. **(G)** The results obtained using the spectrofluorometer are indicated in relative fluorescence units (RFU) emitted by the endothelial monolayer without monocytes and with monocytes attached without stimulus or baseline adhesion and with LPS. **(H)** IgG from patients with RI + and β2GPI increased the adhesion of monocytes compared with IgG from patients with VTI/aPL- and VTII/aPL-. A two-way ANOVA and the Holm–Sidak post-test (*p < 0.05) were performed. The results were obtained from three independent experiments. The arrow indicates the aggregates of monocytes. PM/VT, pregnancy morbidity and vascular thrombosis.

Monocyte aggregates were not observed in the endothelium regarding basal adhesion and cells treated with VTI/aPL, VTII/aPL, and NHS controls (Figures 2A,C–E). As expected, LPS increased the adhesion of THP-1 monocytes to an endothelial monolayer (Figures 2B,G). Complementarily, RI+ IgG increased the adhesion of monocytes to the endothelium compared with VTI/aPL and VTII/aPL controls (Figures 2D–F,H). The number of aggregates was higher and statistically significant upon RI+ stimulation (Figures 2F,H).

### Immunoglobulin-G From Patients With RII+ Induced an Augmentation in O_2_^–^ Production

**FIGURE 3:**
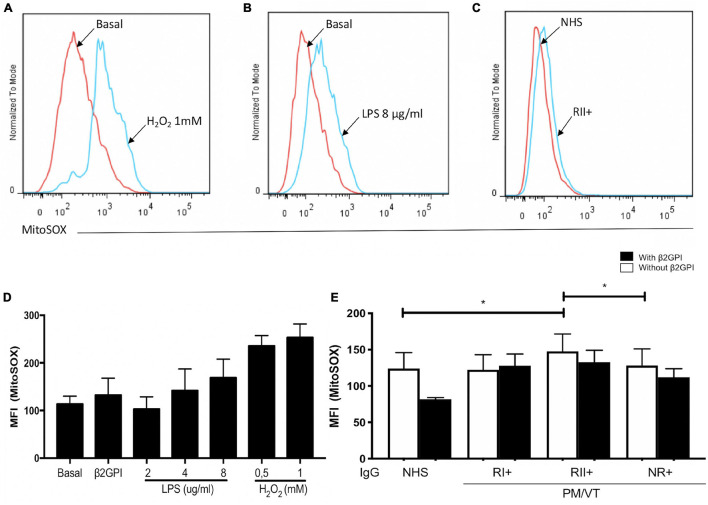
Immunoglobulin-G from patients with refractory and secondary obstetric APS (RII+) induced O_2_^–^ mitochondrial production. **(A,B,D)** Hydrogen peroxide and LPS increased the MFI of MitoSOX in HUVECs. **(C,E)** RII + IgG group induced mitochondrial O_2_^–^ production in HUVECs without β2GPI compared with the IgG from NHS and non-refractory primary obstetric APS (NR+) groups. A two-way ANOVA and the Holm–Sidak post-test (*p < 0.05) were performed. The results were obtained from five independent experiments. **(A–C)** Representative histograms. PM/VT, pregnancy morbidity and vascular thrombosis.

Hydrogen peroxide and LPS increased the endothelial O_2_^–^ production (Figures 3A,B,D). RII+ IgG increased endothelial O_2_^–^ production compared with the NHS and NR+ IgG without β2GPI (Figures 3C,E). No IgG effect was observed in cells treated with β2GPI (Figure 3E). In addition, IgG from the patient groups included in this study and β2GPI alone did not affect the ROS production detected by DCF signal or lipoperoxidation by BODIPY C11 staining (Supplementary Figure 1). IgG from patients with positive or negative aPL and thrombosis (VTI/aPL*-*, VTII/aPL*-*, VTI, and VTII) did not affect the generation of oxidative stress in HUVECs (data not shown).

### Immunoglobulin-G From Patients With NR+ and RII+ Increased the Cell-Derived Endothelial Microparticles

**FIGURE 4:**
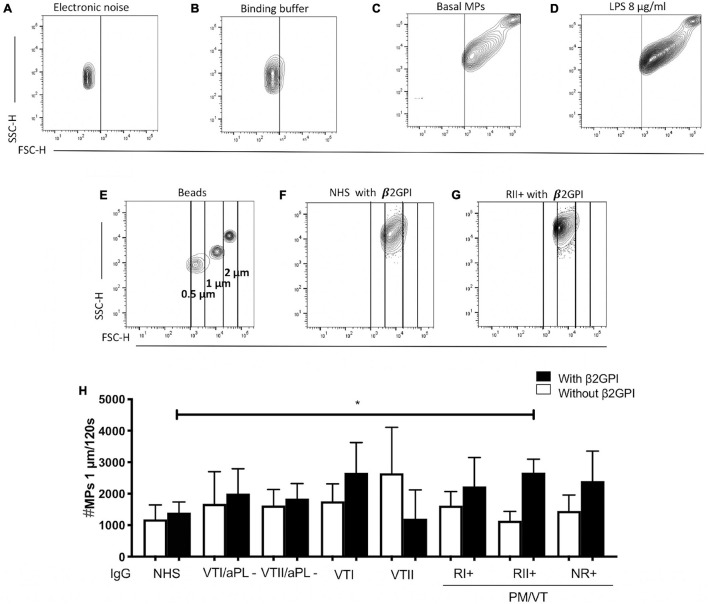
Immunoglobulin-G from patients with secondary refractory APS (RII+) increased the number of 1-μm microparticles (MPs). **(A)** Electronic noise was excluded from all the analyses. **(B)** The binding buffer for annexin V staining did not present a significant number of events. **(C,D)** LPS stimulation increased the number of MPs compared with no stimulation. **(E)** For all the analyses, the approximate size of the MPs was delimited with 0. 5-, 1-, and 2-μm beads. **(F–H)** IgG of patients with RII + induced the generation of 1-μm MPs in a β2GPI-dependent manner compared with IgG from NHS control. A two-way ANOVA and the Holm–Sidak post-test (*p < 0.05) were performed. The results were obtained from three independent experiments. PM/VT, pregnancy morbidity and vascular thrombosis.

**FIGURE 5:**
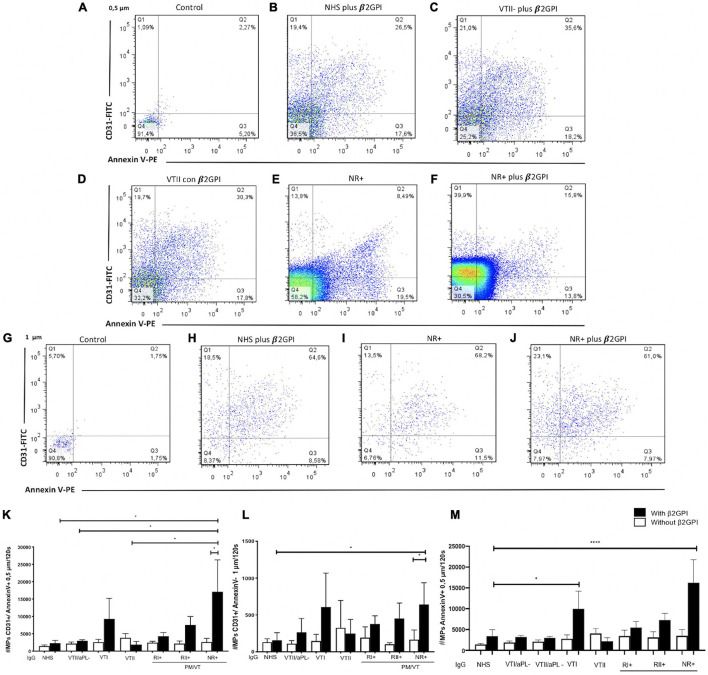
Immunoglobulin-G from patients with non-refractory primary obstetric APS (NR+) induced the production of 0.5- and 1-μm endothelial MPs. **(A,G)** Control indicates MPs without antibodies, which were used to define the location of the negative and positive MPs for Annexin V and CD31. **(A–F,K)** IgG/NR+ increased the number of 0.5-μm CD31+/annexin V+ MPs in the presence of β2GPI, compared with NHS IgG, secondary thrombotic APS (VTII)/antiphospholipid antibodies (aPL) IgG, VTII IgG, and IgG of patients with NR+ without β2GPI. **(G–J,L)** IgG from patients with NR+ increased the number of MPs of 1-μm CD31+/annexin V- in a β2GPI-dependent manner compared with the IgG/NHS control and patients with NR+ without β2GPI. **(M)** IgG from patients with VTI and NR+ increased the number of total MPs of 0.5-(μm annexin V (in a (β2GPI-dependent manner more than IgG/NHS; Annexin V (MPs are the indicators of procoagulant potential. A two-way ANOVA and the Holm–Sidak post-test (*p < 0.05) were performed. The results were obtained from three independent experiments. PM/VT, pregnancy morbidity and vascular thrombosis. ****p < 0.0001.

A higher number of 0.5- and 1-μm endothelial microparticles for CD31+/annexin V***-***, CD31+/annexin V+, CD31-/annexin V+, and CD31-/annexin ***V-*** were generated in cells stimulated with LPS compared with basal microparticle production (Supplementary Figures 2A–N). After the initial set up of an experimental protocol, including a potential experimental confounder generated by electronic noise or negligible count of particles in the binding buffer (Figures 4A,B) and using LPS as a positive control to increase the total microparticle count (Figures 4C,D), the effect of IgG from patients with APS was tested. In addition, the microparticle size was estimated using the polystyrene beads of 0.5, 1, and 2 μm (Figure 4E). Contrarily, NR+ IgG with β2GPI increased the generation of 0.5-μm CD31+/annexin V+ microparticles compared with IgG from the same group without β2GPI and NHS IgG, VTII/aPL*-*, and VTII controls with β2GPI (Figures 5B–F,K). IgG from this group with β2GPI increased the generation of 1-μm CD31+/annexin ***V-*** microparticles compared with IgG without β2GPI or NHS control IgG (Figures 5H–J,L). RII+ IgG increased the production of 1-μm endothelial dysfunction biomarkers of the total cell-derived endothelial microparticles. RII+ IgG increased the generation of microparticles (1 μm) in a β2GPI-dependent manner compared with the NHS control (Figures 4F–H).

### Immunoglobulin-G From Patients With NR+ and Primary Thrombotic Antiphospholipid Syndrome Induced Production of Procoagulant (Annexin V+) Cell-Derived Endothelial Microparticles

Non-refractory primary obstetric APS and VTI IgG increased the procoagulant microparticles or Annexin V+ (CD31+/annexin V+ and CD31-/annexin V+) by 0.5 μm (Figure 5M). IgG from the patient groups included in this study did not induce 2-μm microparticles (data not shown). The dot-plot labeled with control indicates microparticles without antibodies, which were used to define the location of negative and positive MPs for Annexin V and CD31 (Figures 5A,G).

### All Immunoglobulin-G From Patients With Pregnancy Morbidity and Thrombosis (RI+, RII+, and NR+) Induced Endothelial Dysfunction Through a Decreased Nitric Oxide Production in Human Umbilical Vein Endothelial Cells

**FIGURE 6:**
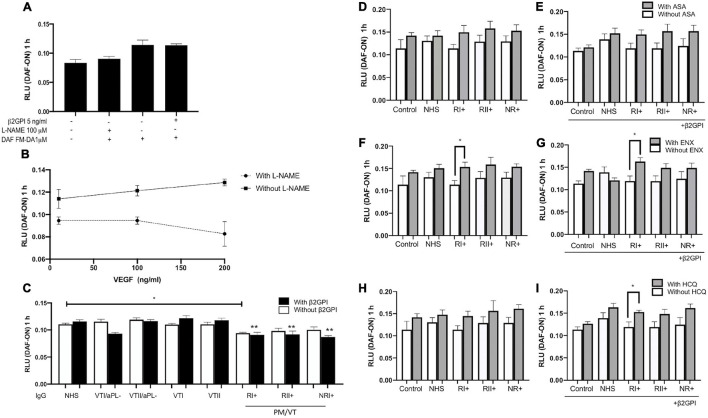
All IgG from patients with pregnancy morbidity and thrombosis (RI+, RII+, and NR+) induced endothelial dysfunction through a decrease in nitric oxide (NO) production in HUVECs. **(A)** β2GPI alone did not alter NO production in contrast with the basal control. **(A,B)** NO release is reduced by the addition of NG-nitro-L-arginine methyl ester (L-NAME). **(B)** Vascular endothelial growth factor (VEGF) induced NO production in a dose-dependent manner in contrast with cells treated with L-NAME, which exerted its antagonistic effect. **(C)** IgG/RI+ decreased NO bioavailability in contrast with the IgG/NHS control without β2GPI. All IgG from patients with pregnancy morbidity and thrombosis (RI+, RII+, and NR+) plus β2GPI reduced NO production compared with the IgG/NHS control (**p < 0.01). A two-way ANOVA and the Holm–Sidak post-test (*p < 0.05) were performed. **(D,E)** Aspirin (ASA) had no modulatory effect on the reduction of NO induced by IgG. **(F,G)** Enoxaparin (ENX) restored NO bioavailability reduced by IgG/RI+ with and without β2GPI. **(H,I)** Hydroxychloroquine (HCQ) restored NO bioavailability reduced by IgG/RI+ with β2GPI. A *t*-test (*p < 0.05) was performed in ASA and EXN analyses. The HCQ data were analyzed using the Mann–Whitney test (*p < 0.05). The results were obtained from four independent experiments. PM/VT, pregnancy morbidity and vascular thrombosis.

Vascular endothelial growth factor-induced NO synthesis in a dose-dependent manner, whereas L-NAME inhibited its production in all the VEGF-used doses (Figures 6A,B). β2GPI alone did not alter NO synthesis in contrast with the baseline control (Figure 6A). RI+ IgG decreased NO synthesis in HUVECs compared with NHS control IgG without β2GPI (Figure 6C). All IgG from patients with pregnancy morbidity and thrombosis (RI+, RII+, and NR+) reduced NO synthesis compared with the NHS IgG control in a β2GPI-dependent manner (Figure 6C). ASA did not induce a modulatory effect on reducing NO induced by IgG from patients with pregnancy morbidity and thrombosis (Figures 6D,E). ENX restored the reduced synthesis of NO generated by RI+ IgG with and without β2GPI (Figures 6F,G). HCQ did not induce a modulatory effect on reducing NO induced by IgG from patients with pregnancy morbidity and thrombosis without β2GPI (Figure 6I). HCQ restored the synthesis of NO reduced by IgG from RI+ with β2GPI (Figure 6H). IgG, ASA, EXN, and HCQ did not induce dead cells compared with baseline control (Supplementary Figure 3).

### All Immunoglobulin-G From Patients With Antiphospholipid Syndrome Induced Endothelial Dysfunction Through an Alteration in Endothelin-1 or Vascular Endothelial Growth Factor Production in Human Umbilical Vein Endothelial Cells Supernatants

**FIGURE 7:**
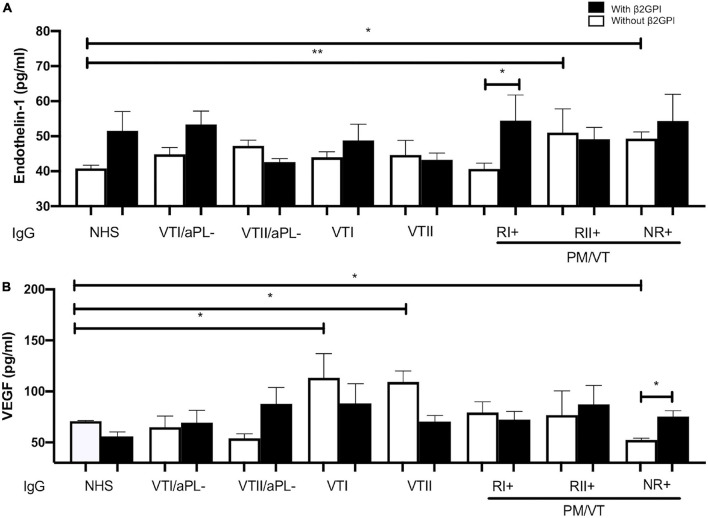
All IgG from patients with APS-induced endothelial dysfunction through an alteration in endothelin-1 or VEGF production in HUVEC supernatants. **(A)** RII+ and NRI+ IgG groups of patients increased endothelin-1 production compared with IgG/NHS without β2GPI. RI+ IgG with β2GPI increased the production of endothelin-1 compared with this same group without β2GPI. **(B)** IgG from patients with VTI and VTII increased the VEGF in contrast with NHS IgG without β2GPI. IgG from patients with NR+ decreased the VEGF amount in contrast with NHS IgG without β2GPI. NR+ IgG and β2GPI increased the VEGF production compared with this same IgG without β2GPI. A two-way ANOVA and the Holm–Sidak post-test (*p < 0.05; **p < 0.01) were performed. The results were obtained from three independent experiments. PM/VT, pregnancy morbidity and vascular thrombosis.

Refractory secondary obstetric APS and NR+ IgG increased the presence of endothelin-1 compared with NHS IgG without the addition of β2GPI (Figure 7A). RI+ IgG with β2GPI increased the production of endothelin-1 compared with RI+ IgG without β2GPI (Figure 7A). IgG from patients with VTI and VTII increased the VEGF in contrast with NHS IgG in the absence of β2GPI. IgG from patients with NR+ decreased the VEGF levels in contrast with IgG from the NHS in the absence of β2GPI. NR+ IgG with β2GPI increased VEGF levels compared with that without β2GPI (Figure 7B).

## Discussion

### Endothelial Activation and Dysfunction in Antiphospholipid Syndrome: Association With Clinical Manifestations

**FIGURE 8:**
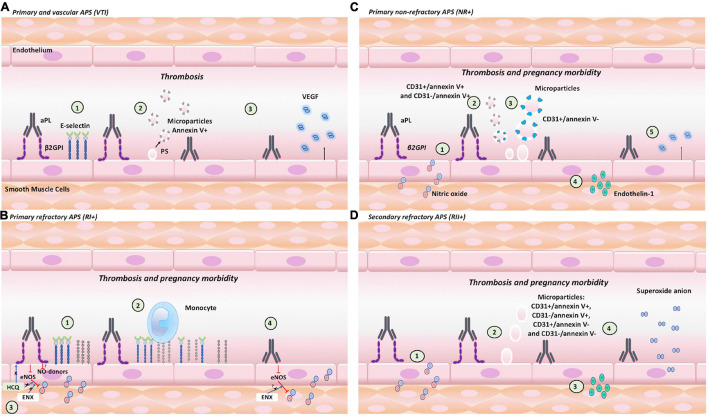
Endothelial activation and dysfunction in APS: clinical manifestation association and role of β2GPI. The pathogenetic effects associated with endothelial activation induced by aPL with or without β2GPI contribute to thrombosis or pregnancy morbidity. **(A)** *VTI*+ *IgG:* (1) increased the expression of E-selectin; (2) generated positive annexin V procoagulant MPs of 0.5 μm; and (3) increased the VEGF generation. **(B)** *RI*+ *IgG:* (1) increased the expression of E-selectin and VCAM; (2) induced monocyte adhesion; (3) decreased NO bioavailability, which was modulated by HCQ and ENX; and (4) decreased NO bioavailability, which was modulated by ENX. **(C)** *NR*+ *IgG:* (1) decreased NO bioavailability; (2) increased the number of 0.5-μm procoagulant MPs (CD31+/annexinV+ and CD31-/annexinV+); (3) increased CD31+/annexinV, MP of 1 μm; (4) increased endothelin-1 production; and (5) reduced VEGF. **(D)** *RII*+ *IgG:* (1) decreased NO bioavailability; (2) increased MPs of 1 μm; (3) generated endothelin-1 production; and (4) induced oxidative stress (mitochondrial O_2_^–^ production).

Different endothelial activation and dysfunction mechanisms induced by IgG in groups of patients included in our study are associated with the development of APS clinical manifestations. Our main findings include: (1) VTI IgG increased the expression of E-selectin, generated positive procoagulant microparticles, and increased VEGF generation (Figure 8A). (2) RI+ IgG increased the adhesion of monocytes and decreased NO, which were modulated by HCQ and ENX (Figure 8B). The results suggest that HCQ and ENX together become an effective alternative for these patients, considering the endothelial dysfunction modulation induced by the previous activation of these cells. (3) NR+ IgG decreased NO bioavailability, increased procoagulant microparticles, induced endothelin-1, and reduced VEGF production (Figure 8C). In these NR+ patients, heparin and ASA could modulate the clinical manifestations using the mechanisms that were not evaluated in this study. (4) IgG from RII+ decreased NO bioavailability, generated the production of microparticle and endothelin-1, and induced oxidative stress (mitochondrial O_2_^–^ production) (Figure 8D). In these groups of patients, the antioxidant therapy, which was proposed by a few authors, was used for APS treatment.

Both the aPL that require or do not require β2GPI to increase their pathological effect on the endothelium could act synergistically in each group of patient to induce clinical manifestations. aPL are associated with different clinical manifestations of APS, but the mechanism that explains these associations or the presence of these autoantibodies or epitopes in distinct groups of patients with APS is unknown, especially in the refractory cases. aPL generate endothelial activation leading to cell dysfunction and favor clinical manifestations of thrombosis and gestational morbidity. These aPL recognize antigens, such as cardiolipin and β2GPI, and epitopes of these antigens that determine the pathological effect or clinical manifestation. An example of this is the antibodies anti-domain-I of β2GPI (aD1-β2GPI) in the glycine40-arginine 43 associated with thrombosis (de Laat et al., 2005). However, the presence of these aD1-β2GPI is a predictor of thrombosis and pregnancy morbidity; contrarily, the antibodies anti-domain-4/5 of β2GPI is not associated with these clinical manifestations (Chighizola et al., 2018). Additionally, patients with SLE, LA, and aβ2GPI IgA antibodies are also associated with developing thrombosis and aCL with preterm delivery (Saleh et al., 2020; Demir et al., 2021). Patients with different clinical manifestations, refractoriness, and other autoimmune diseases, such as SLE, were included to differentiate between the pathological mechanisms of aPL on endothelial activation and dysfunction that explain the aPL generation of different pathological effects and triggering thrombosis alone or thrombosis with pregnancy morbidity.

### Proadhesive Phenotype as an Indicator of Endothelial Activation Leading to Endothelial Dysfunction

Immunoglobulin-G from the different groups of patients triggered different mechanisms to generate endothelial activation and dysfunction that explain APS clinical manifestations. RI+ IgG with β2GPI induced endothelial activation by expressing E-selectin, VCAM, and consequent monocyte adhesion to the endothelium. Both molecules increased by aPL indicate endothelial activation associated with thrombus formation (Gandhi et al., 2021). In our experimental model, VTI IgG did not induce endothelial dysfunction as evaluated with NO synthesis. RI+ IgG induced endothelial activation and dysfunction. RI+ IgG induced endothelial dysfunction due to the NO bioavailability reduction with and without β2GPI. The expression of adhesion molecules is mediated by the factor NF-kB that is inhibited by NO (Liao, 2013). NO produced by eNOS or NO-donors reduces endothelial activation by inhibiting the expression of adhesion molecules, leukocyte adhesion and traffic, platelet reactivity, and vascular proliferation and angiogenesis modulation (Liao, 2013). The expression of adhesion molecules allowing the binding of monocytes to the endothelium and the decreased NO were associated with thrombosis (Ghimire et al., 2017). Monocytes are the primary sources of tissue factor, a key in the extrinsic coagulation cascade. These cells also bind platelets to each other and participate in thrombus recanalization (Mukhopadhyay et al., 2019). On the other hand, NO reduction represents a prothrombotic microenvironment considering that NO inhibits platelet and leukocyte adhesion to the endothelium (Costa et al., 2019). These mechanisms of endothelial activation and dysfunction induced by RI+ IgG associated with thrombosis also lead to gestational morbidity (Possomato-Vieira and Khalil, 2016). Therefore, decreased NO was associated with increased vasoconstriction and hypertension, leading to an alteration in adequate uterine spiral artery remodeling (Possomato-Vieira and Khalil, 2016). In addition, similar monocyte-mediated activation of endothelial cells was described in preeclampsia, a pregnancy condition characterized by hypertension, endothelial dysfunction, and, in severe cases, thrombocytopenia.

In APS, the development of thrombosis is associated with reduced endothelial NO through the binding of aPL to the apolipoprotein E receptor 2 (ApoER2) by β2GPI, which leads to the protein phosphatase 2A (PP2A) activation that induces eNOS dephosphorylation (Sacharidou et al., 2018). Recently, the same proteins were detected in trophoblast with pathological functions, leading to pregnancy morbidity. The binding of aPL by ApoER2-stimulated PP2A, which reduced proliferation and trophoblastic migration associated with the development of preeclampsia for upregulation in hypoxia-inducible factor 1 and soluble endoglin (Chu et al., 2021). In APS, the development of pregnancy morbidity is related to endothelial activation and dysfunction that induce defective placental formation; however, the effect of aPL on trophoblast cells is vital (Abrahams et al., 2017; Quao et al., 2018). aPL reduce decidual endovascular trophoblast invasion (Sebire et al., 2002), leading to early recurrent miscarriages in APS (Abrahams et al., 2017; Jovanovic Krivokuca et al., 2017; Quao et al., 2018). Contrarily, aPL also induce pregnancy morbidity in the second and third trimester by inducing placental dysfunction that is evidenced by preeclampsia and/or intrauterine growth restriction (Antovic et al., 2018; Chu et al., 2021).

### Hydroxychloroquine and Enoxaparin Restored Nitric Oxide Reduced by RI+ Immunoglobulin-G

Hydroxychloroquine (HCQ) and ENX modulate the endothelial dysfunction induced by RI+ IgG and β2GPI. ENX only modulated endothelial dysfunction generated by RI + IgG without β2GPI. Miranda et al. (2019) showed that HCQ restores eNOS phosphorylation decreased by aPL and thrombus formation *in vivo*. These authors found that HCQ reverses the increased tissue factor and decreased thrombomodulin induced by aPL in human aortic endothelial cells (Miranda et al., 2019). However, the previous study did not include different groups of patients, and the effects of HCQ on NO bioavailability through the blockade of NO-donors are unknown. Our study found the existence of the modulating effect of HCQ in patients with refractory primary APS with pregnancy morbidity and thrombosis (RI+). This modulating effect of HCQ is also explained by the disintegration that this drug induces on the phospholipid/β2GPI/aβ2GPI complexes (Rand et al., 2008). HCQ disintegrates the β2GPI/aPL complexes, preventing downstream pathological effects; it also modulates proinflammatory cytokine production, such as tumor necrosis factor-alpha, and mitigates the increase of HUVEC permeability induced by the serum from patients with preeclampsia (Rahman et al., 2020). Likewise, heparin binds to β2GPI in domain V through an interaction with Lys284, Lys286, and Lys287, and decreases the ability to recognize aPL aβ2GPI by reducing their prothrombotic activity (Guerin et al., 2002). Similar to HCQ, heparin increases the phosphorylation of eNOS, thus increasing NO (Li et al., 2020).

### Increased Endothelin-1 and Vascular Endothelial Growth Factor Production Changes as an Indicator for Endothelial Dysfunction Associated With Antiphospholipid Syndrome Clinical Manifestations

Non-refractory primary obstetric APS IgG with β2GPI decreased NO bioavailability and VEGF production but increased endothelin-1 generation, which explains one of the triggering mechanisms of thrombosis and pregnancy morbidity and hypertension. Our results agree with previous findings in which pregnancy morbidity was associated with a decreased NO that induces endothelin-1 production (Saleh et al., 2016). Nevertheless, a VEGF reduction was related with enhancing this vasoconstrictor as a switch in the angiogenic mechanisms, associated with placental malformation, pregnancy morbidity, and other pathologies, such as cancer (Lankhorst et al., 2016). Despite this, IgG from patients with VTI and VTII increased the VEGF production as a possible thrombosis-inducing mechanism associated with intimal hyperplasia at the thrombus site (Williams et al., 2000).

### Induction of Oxidative Stress Associated With Endothelial Dysfunction

On the other hand, RII+ IgG with β2GPI decreased NO bioavailability, but without this cofactor increased endothelin-1 and induced O_2_^–^ production compared with the NR+ primary APS (NR+). Independent results of β2GPI indicate that this effect is generated by aCL without requiring this cofactor or other autoantibodies present in RII+ patients. Human monoclonal aCL injected into BALB/c mice was reported to induce O_2_^–^ production. However, these antibodies showed reactivity to β2GPI (Delgado Alves et al., 2005). Hence, the role of aCL in this event was unclear, but Simoncini et al. (2005) showed that after eliminating aβ2GPI, aPL from patients with APS-induced ROS production in endothelial cells compared with the NHS IgG control, suggesting the relevance of β2GPI-independent aCL. A few studies reported that the serum of patients with SLE, positive for anti-double-stranded DNA antibodies, induced intracellular ROS production and increased NADPH oxidase activity, which catalyzes O_2_^–^ production (Didion and Faraci, 2002; Toral et al., 2017). However, patients were not classified by clinical manifestation in these previous studies. According to the results in this study, β2GPI-independent autoantibodies from patients with RII+ trigger thrombosis and pregnancy morbidity through endothelial oxidative stress, indicating a potential antioxidant therapy in patients with refractoriness to conventional treatment. These results indicate that not only β2GPI-independent aCL generate events associated with endothelial activation in these patients. RII+ IgG triggers endothelial activation and dysfunction, characterized by ROS production: O_2_^–^ production reduces NO bioavailability, impairing vascular relaxation, which increases in endothelin-1 and enhances vasoconstriction (Silva et al., 2012).

### Immunoglobulin-G From Patients With NR+ Obstetric Antiphospholipid Syndrome-Induced Endothelial Microparticles Production, Increasing Endothelial Activation and Dysfunction

Endothelial dysfunction is characterized by the outburst of microparticles (Amabile et al., 2005; Deng et al., 2017; Leite et al., 2020). IgG from patients with APS, presenting thrombosis with or without pregnancy morbidity, increased endothelial microparticles *in vitro* (Pericleous et al., 2013). Endothelial microparticles reportedly increased only in the plasma of patients with thrombotic APS compared with healthy controls and patients with obstetric APS (Breen et al., 2015). VTI IgG with β2GPI produced 0.5-μm microparticles positive for phosphatidylserine, considered as procoagulants. These microparticles induce coagulation by exposing phosphatidylserine, which activates tissue factor, a receptor for factor VIIa, and initiates the coagulation cascade through an extrinsic pathway (Owens and Mackman, 2011). Additionally, NR+ IgG increased the number of 0.5-μm procoagulant microparticles (CD31+/annexinV+ and CD31-/annexinV+) and a 1-μm CD31+ microparticle. RII+ IgG generates total microparticles of 1 μm. Microparticles of endothelial cells were found in patients with recurrent miscarriage, indicating damage and endothelial activation (Carp et al., 2004).

## Conclusion

Our results suggest that endothelial activation and dysfunction in APS are seen in different contexts, and the induction mechanism varies according to the clinical characteristics of patients and the presence of aPL cofactors, such as β2GPI. HCQ only modulates endothelial dysfunction generated by RI+ IgG with β2GPI, and ENX modulates endothelial dysfunction generated by RI+ IgG with or without β2GPI. Additionally, we recommended that in future studies, patients with APS must be classified according to clinical manifestation and that these findings should be considered when using these drugs in patients with refractory APS, specifically primary (RI+).

## Strengths and Limitations

In this study, the classification of patients by clinical manifestations and the addition of β2GPI to the HUVECs allowed us to understand the differences that aPL present in endothelial activation and dysfunction, representing an adequate methodology in the study of APS. However, the number of individuals in this study was limited, considering the difficulty in recruiting patients with autoimmunity well-characterized by the low prevalence of this type of disease even more with refractoriness. Our experimental design should be applied to a larger population of patients.

## Data Availability Statement

The raw data supporting the conclusions of this article will be made available by the authors, without undue reservation.

## Ethics Statement

The studies involving human participants were reviewed and approved by the Ethics Committee of the University of Antioquia. The patients/participants provided their written informed consent to participate in this study.

## Author Contributions

ÁC and MV generated the conception of this study. MV wrote the draft of the manuscript, performed the experiments, and analyzed the data. LP performed some experiments on expression adhesion molecules. MR, SS, RN-S, CE, JV, and ÁC contributed to the analysis and interpretation of data. All authors have reviewed and approved the manuscript.

## Conflict of Interest

The authors declare that the research was conducted in the absence of any commercial or financial relationships that could be construed as a potential conflict of interest.

## Publisher’s Note

All claims expressed in this article are solely those of the authors and do not necessarily represent those of their affiliated organizations, or those of the publisher, the editors and the reviewers. Any product that may be evaluated in this article, or claim that may be made by its manufacturer, is not guaranteed or endorsed by the publisher.

## Abbreviations

APS: antiphospholipid syndrome
aPL: antiphospholipid antibodies
β 2GPI: beta 2-glycoprotein-I
IgG: immunoglobulin-G
VTI and VTII: primary and secondary thrombotic APS
RI+: refractory primary obstetric APS
NR+: non-refractory primary obstetric APS
RII+: refractory secondary obstetric APS
VTI/aPL−: patients negative for aPL with thrombosis without autoimmune disease
VTII/aPL−: patients negative for aPL with thrombosis and SLE
NHS: normal human serum
NO: nitric oxide
VEGF: vascular endothelial growth factor
LA: lupus anticoagulant
aCL: anti-cardiolipin antibodies
a β 2GPI: anti- β 2-glycoprotein-I
HCQ: hydroxychloroquine
ROS: reactive oxygen species
VCAM-1: vascular cell adhesion molecule 1
NOS: nitric oxide synthase
MCP-1: monocyte chemoattractant protein I
FBS: fetal bovine serum
DCFH-DA: dichlorofluorescein diacetate
L-NAME: arginine methyl ester
DAF-FM-DA, 4-amino-5-methylamino-2′: 7-difluoroflurescein diacetate
ENX: enoxaparin
ASA: aspirin
7-AAD: 7-aminoactinomycin D
HUVECs: human umbilical vein endothelial cells
MFI: median fluorescence intensity
FMO: fluorescence-minus-one.
