# Supplementary material for: Differences in Endothelial Activation and Dysfunction Induced by Antiphospholipid Antibodies Among Groups of Patients With Thrombotic, Refractory, and Non-refractory Antiphospholipid Syndrome
Source: Front Physiol. 2021 Dec 2;12:764702. doi: 10.3389/fphys.2021.764702 (PMC8675389; doi:10.3389/fphys.2021.764702)
Supplement: Supplementary file 1 [file Data_Sheet_1.DOCX]

Supplementary Material


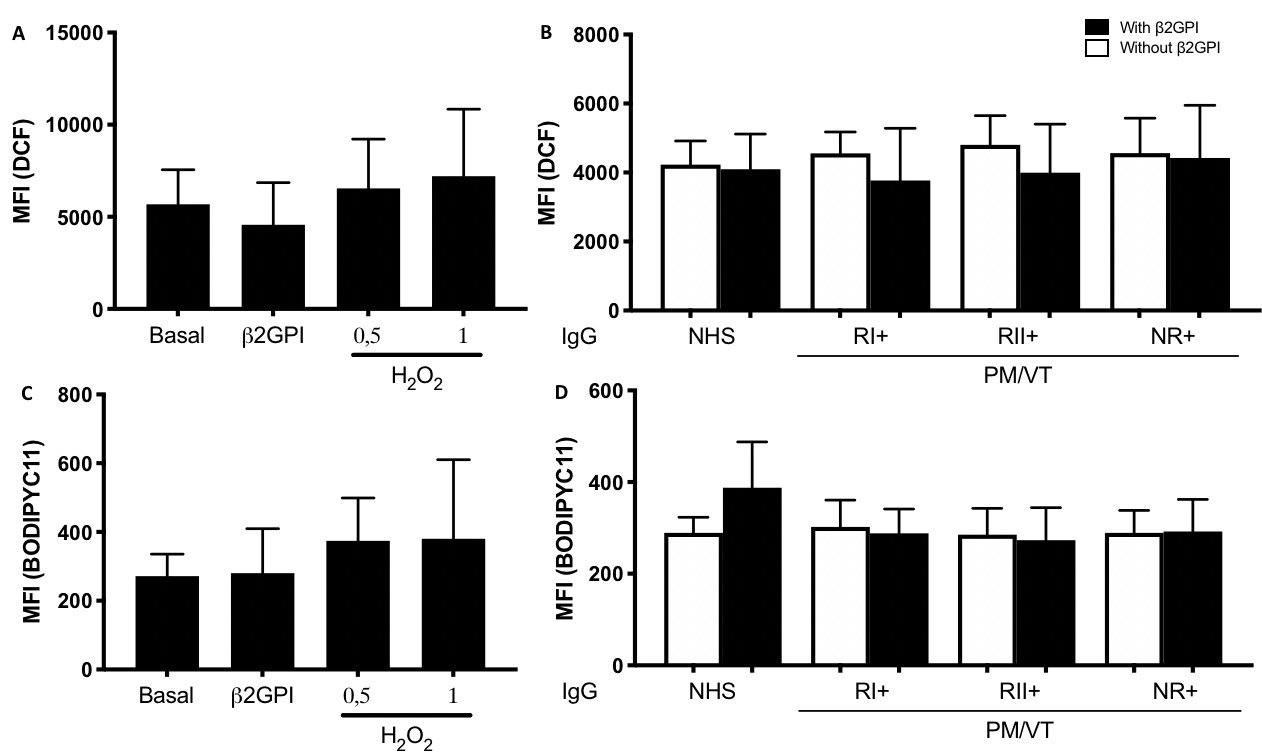


**Supplementary Figure 1. ROS production and lipoperoxidation in HUVECs.** (A and C) Hydrogen peroxide (H_2_O_2_) increased the median fluorescence intensity (MFI) of MitoSOX and BODIPYC11 in HUVECs, whereas β2GPI alone did not affect ROS production. (A-D) IgG from groups of patients included in the study did not affect the intracellular ROS production detected by DCF signal or lipoperoxidation by BODIPY C11 staining**.** Results were obtained from three independent experiments. PM/VT indicates patients with pregnancy morbidity and vascular thrombosis. The IgG from women with positive or negative thrombosis did not affect the generation of oxidative stress in HUVECs (data not shown).


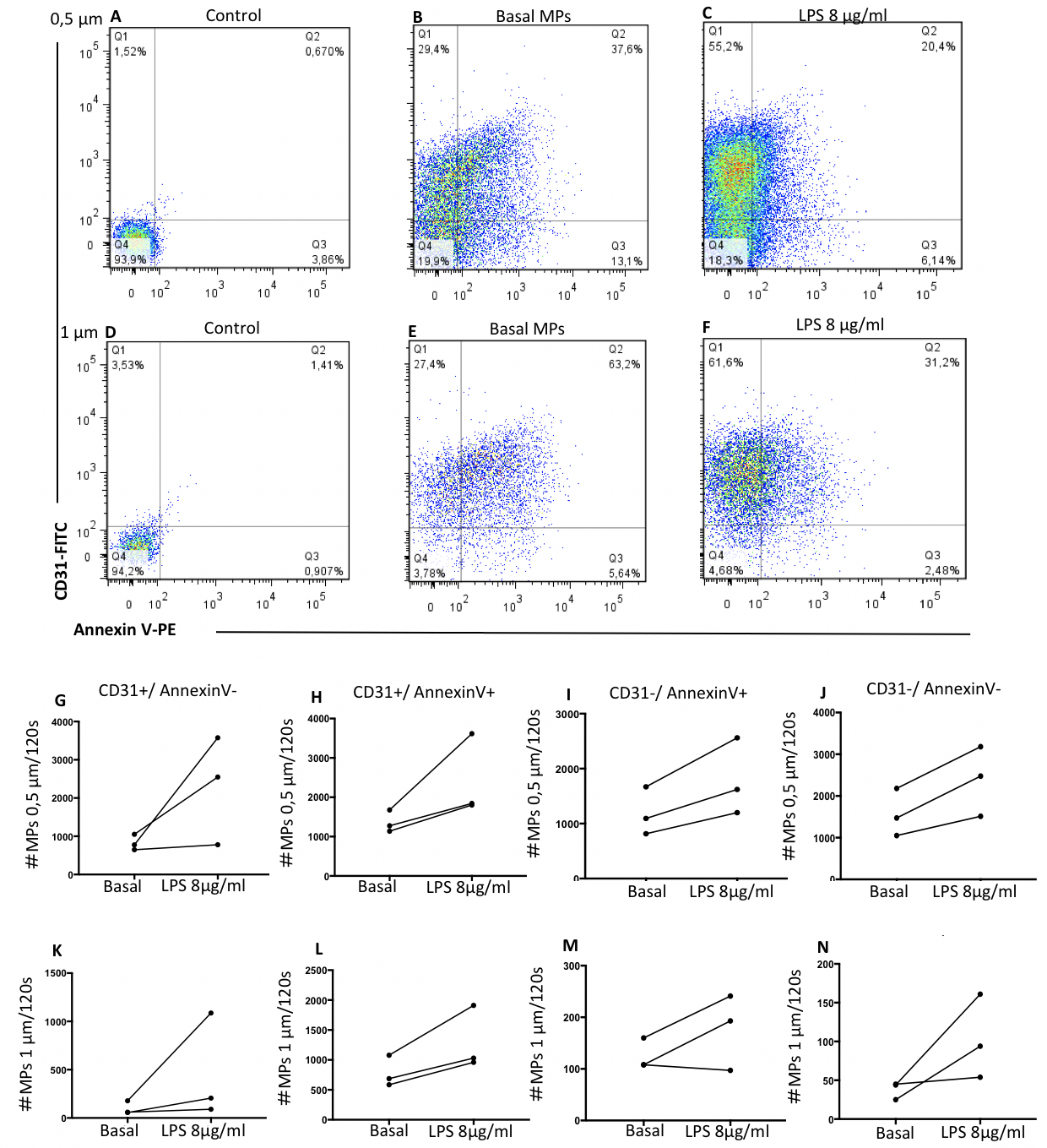


**Supplementary Figure 2. Evaluation of cell-derived endothelial microparticles (MPs)**. (A and D) Control indicates microparticles without antibodies, which were used to define the location of the negative and positive MPs for Annexin V and CD31. Lipopolysaccharide (LPS) increased CD31+/annexin V-, CD31+/annexin V+, CD31-/annexin V+, and CD31-/annexin V- MPs of 0.5 μm (A–C and G–J) and 1 μm (D–F and K–N) compared with baseline production. Results were obtained from three independent experiments.


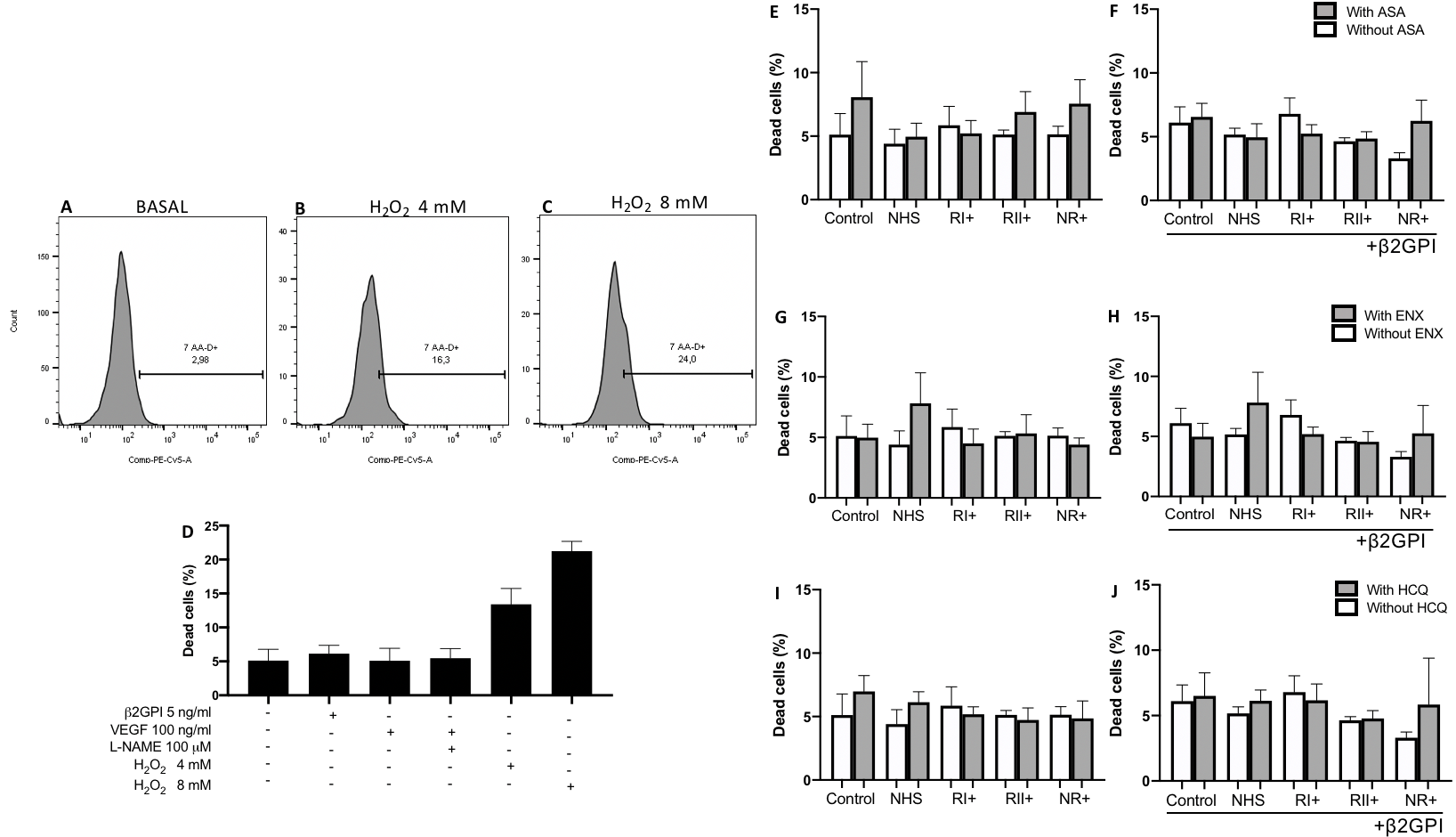


Supplementary Figure 3. Effect of IgG, aspirin (ASA), enoxaparin (ENX), and hydroxychloroquine (HCQ) on cell viability. (A–D) 4 and 8 mM of hydrogen peroxide (H_2_O_2_) increased the dead cells compared with baseline control, beta 2-glycoprotein-I (β2GPI), vascular endothelial cell growth factor (VEGF), and NG-nitro-L-arginine methyl ester (L-NAME) in HUVECs. (E–J) IgG, ASA, EXN, and HCQ did not induce dead cells compared with baseline control. Results were obtained from three independent experiments.
